# Characterization of the 1S–2S transition in antihydrogen

**DOI:** 10.1038/s41586-018-0017-2

**Published:** 2018-04-04

**Authors:** M. Ahmadi, B. X. R. Alves, C. J. Baker, W. Bertsche, A. Capra, C. Carruth, C. L. Cesar, M. Charlton, S. Cohen, R. Collister, S. Eriksson, A. Evans, N. Evetts, J. Fajans, T. Friesen, M. C. Fujiwara, D. R. Gill, J. S. Hangst, W. N. Hardy, M. E. Hayden, C. A. Isaac, M. A. Johnson, J. M. Jones, S. A. Jones, S. Jonsell, A. Khramov, P. Knapp, L. Kurchaninov, N. Madsen, D. Maxwell, J. T. K. McKenna, S. Menary, T. Momose, J. J. Munich, K. Olchanski, A. Olin, P. Pusa, C. Ø. Rasmussen, F. Robicheaux, R. L. Sacramento, M. Sameed, E. Sarid, D. M. Silveira, G. Stutter, C. So, T. D. Tharp, R. I. Thompson, D. P. van der Werf, J. S. Wurtele

**Affiliations:** 1 Department of Physics, University of Liverpool, Liverpool, UK; 2 Department of Physics and Astronomy, Aarhus University, Aarhus, Denmark; 3 Department of Physics, College of Science, Swansea University, Swansea, UK; 4 School of Physics and Astronomy, University of Manchester, Manchester, UK; 5 Cockcroft Institute, Sci-Tech Daresbury, Warrington, UK; 6 TRIUMF, 4004 Wesbrook Mall, Vancouver, British Columbia Canada; 7 Department of Physics, University of California at Berkeley, Berkeley, CA USA; 8 Instituto de Fisica, Universidade Federal do Rio de Janeiro, Rio de Janeiro, Brazil; 9 Department of Physics, Ben-Gurion University of the Negev, Beer-Sheva, Israel; 10 Department of Physics and Astronomy, University of Calgary, Calgary, Alberta Canada; 11 Department of Physics and Astronomy, University of British Columbia, Vancouver, British Columbia Canada; 12 Department of Physics, Simon Fraser University, Burnaby, British Columbia Canada; 13 Department of Physics, Stockholm University, Stockholm, Sweden; 14 Department of Physics and Astronomy, York University, Toronto, Ontario Canada; 15 Department of Physics and Astronomy, University of Victoria, Victoria, British Columbia Canada; 16 Department of Physics and Astronomy, Purdue University, West Lafayette, IN USA; 17 Soreq NRC, Yavne, Israel; 18 Physics Department, Marquette University, Milwaukee, WI USA; 19 IRFU, CEA/Saclay, Gif-sur-Yvette Cedex, France

**Keywords:** Experimental particle physics, Exotic atoms and molecules

## Abstract

In 1928, Dirac published an equation^1^ that combined quantum mechanics and special relativity. Negative-energy solutions to this equation, rather than being unphysical as initially thought, represented a class of hitherto unobserved and unimagined particles—antimatter. The existence of particles of antimatter was confirmed with the discovery of the positron^2^ (or anti-electron) by Anderson in 1932, but it is still unknown why matter, rather than antimatter, survived after the Big Bang. As a result, experimental studies of antimatter^3–7^, including tests of fundamental symmetries such as charge–parity and charge–parity–time, and searches for evidence of primordial antimatter, such as antihelium nuclei, have high priority in contemporary physics research. The fundamental role of the hydrogen atom in the evolution of the Universe and in the historical development of our understanding of quantum physics makes its antimatter counterpart—the antihydrogen atom—of particular interest. Current standard-model physics requires that hydrogen and antihydrogen have the same energy levels and spectral lines. The laser-driven 1S–2S transition was recently observed^8^ in antihydrogen. Here we characterize one of the hyperfine components of this transition using magnetically trapped atoms of antihydrogen and compare it to model calculations for hydrogen in our apparatus. We find that the shape of the spectral line agrees very well with that expected for hydrogen and that the resonance frequency agrees with that in hydrogen to about 5 kilohertz out of 2.5 × 10^15^ hertz. This is consistent with charge–parity–time invariance at a relative precision of 2 × 10^−12^—two orders of magnitude more precise than the previous determination^8^—corresponding to an absolute energy sensitivity of 2 × 10^−20^ GeV.

## Abstract (web summary)

The shape of the spectral line and the resonance frequency of the 1S–2S transition in antihydrogen agree very well with those of hydrogen.

## Main

The transition of interest here, between the ground state and the first excited state of antihydrogen, has an energy of about 10.2 eV. The frequency of this transition in hydrogen has been measured^8^ to a few parts in 10^15^. We previously demonstrated^7^ the existence of the transition in antihydrogen, localizing the frequency to a few parts in 10^10^. Here we characterize the spectral line shape of the transition to the limits of precision of our current apparatus.

**Fig. 1:**
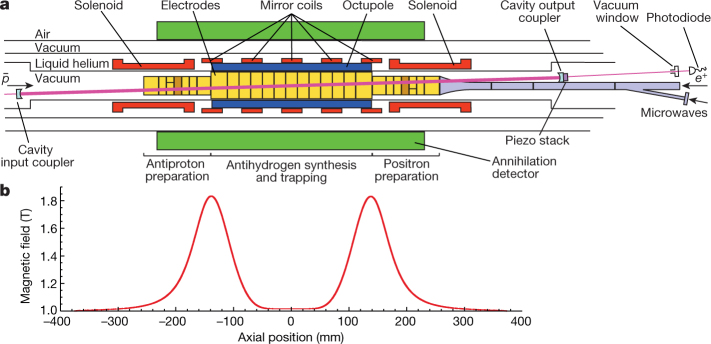
The ALPHA-2 central apparatus and magnetic field profile. **a**, **b**, Penning traps, comprising stacks of cylindrical electrodes immersed in a uniform axial magnetic field generated by an external solenoid (not shown), are used to confine and manipulate antiprotons ($\bar{p}$) and positrons (*e*^+^) to produce antihydrogen. Cold (less that 0.5 K) anti-atoms can be trapped radially by the octupole field and axially by the magnetic well that is formed by the five mirror coils and plotted in **b**. The 243-nm laser light is injected from the antiproton side (left in **a**) and is aligned and position-stabilized on the fixed optical cavity axis. The laser beam crosses the trap axis at an angle of 2.3°. The piezoelectric actuator behind the output coupler is used to modulate the cavity length to lock the cavity to the laser frequency. The axial scale in **a** and **b** is the same; the radial extent of the annihilation detector is larger than illustrated. The vacuum window and photo-diode are further to the right (by about 1 m) than illustrated. The brown-shaded electrodes are used to apply blocking potentials during the experimental trials to ensure that antiprotons that result from ionization are confined to annihilate in the active volume of the detector^7^.

Matter and antimatter annihilate each other, so antihydrogen must be synthesized and then held in ultrahigh vacuum, in isolation from matter, to be studied. The ALPHA-2 apparatus at CERN (Fig. 1) combines antiprotons from the antiproton decelerator^9^ with positrons from a positron accumulator^10, 11^ to produce and trap^12^ atoms of antihydrogen. Antihydrogen can be trapped in ALPHA-2’s magnetic multipole trap if it is produced with a kinetic energy of less than 0.54 K in temperature units. The techniques that we use to produce antihydrogen that is cold enough to trap are described elsewhere^12–14^. In round numbers, a typical trapping trial in ALPHA-2 involves mixing 90,000 antiprotons with 3,000,000 positrons to produce 50,000 antihydrogen atoms, about 20 of which will be trapped. The anti-atoms are confined by the interaction of their magnetic moments with the inhomogeneous magnetic field. The cylindrical trapping volume for antihydrogen has a diameter of 44.35 mm and a length of 280 mm.

The key to anti-atomic spectroscopy, as developed so far^7, 15, 16^, is to illuminate a sample of trapped antihydrogen atoms with electromagnetic radiation (microwaves or laser photons) that causes atoms to be lost from the trap if the radiation is on resonance with the transition of interest. ALPHA-2’s silicon vertex detector^17^ (Fig. 1) affords us single-atom detection capability for the annihilation events associated with lost antihydrogen atoms or antiprotons that encounter the walls of the apparatus. The silicon vertex detector tracks the charged pions from the antiproton annihilation, and various reconstruction algorithms are used to determine the location (vertex) of each annihilation and to distinguish antiprotons from cosmic-ray background using multivariate analysis^18^ (Methods).

To excite the 1S–2S transition, we use a cryogenic, in vacuo enhancement cavity (Fig. 1) for continuous-wave light from a 243-nm laser system (Methods) to boost the intensity in the trapping volume. Long interaction times are possible, because the anti-atoms have a storage lifetime of at least 60 h in the trap. Two counter-propagating photons can resonantly excite the ground-state atoms to the 2S state. Absorption of a third photon ionizes the atom, leading to loss of the antiproton from the trap. Atoms that decay from the 2S to the 1S state via coupling to the 2P state may also be lost, owing to a positron spin-flip^19^.

**Fig. 2:**
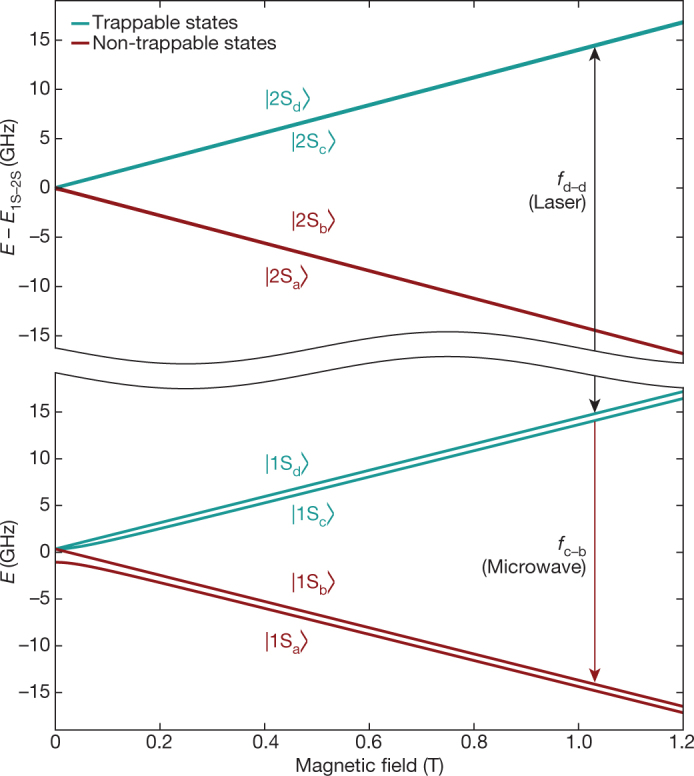
Hydrogenic energy levels. Calculated energies (*E*; for hydrogen) of the hyperfine sublevels of the 1 S (bottom) and 2 S (top) states are plotted against magnetic field strength. The centroid energy difference *E*_1S–2S_ = 2.4661 × 10^15^ Hz has been suppressed on the vertical axis. The vertical black arrow indicates the two-photon laser transition probed here (frequency *f*_d–d_); the red arrow illustrates the microwave transition used to remove the 1S_c_ state atoms (frequency *f*_c–b_).

Referring to the energy-level diagram of hydrogen in Fig. 2, there are two trappable, hyperfine substates of the 1S ground state (labelled ‘c’ and ‘d’). In practice, we find that these states are, on average, equally populated in our trap: *N*_c_ = *N*_d_ = *N*_i_/2, where *N*_i_ is the number of ground-state atoms that are initially trapped in an experimental trial. The 2S state has corresponding hyperfine levels, and we refer to the transitions between the two manifolds as d–d (Fig. 2) and c–c (not pictured).

For each experimental trial, we first accumulate antihydrogen atoms from three mixing cycles or ‘stacks’^13^ and then remove any leftover charged particles using pulsed electric fields. After a wait of about 10 s to allow any excited atoms to decay to the ground state, the trapped population is exposed to laser radiation at a fixed frequency for 300 s. The frequencies used here were chosen to probe only the d–d transition (Fig. 2). Following the laser exposure, we use microwave radiation to remove the 1S_c_ state atoms by driving a resonant spin-flip^15, 16^. The microwave frequency is scanned over 9 MHz in 32 s; these parameters and the injected power level (160 mW at the vacuum feed-through) are chosen to eject anti-atoms quickly while minimizing the perturbation of the vacuum and cryogenic environment. The silicon vertex detector is used to detect annihilations of antihydrogen atoms that are lost during the laser and microwave exposures. Finally, the atom-trap magnets are ramped down in 1.5 s, so that any surviving anti-atoms would be released and their annihilations detected. If the microwave removal of 1S_c_-state atoms is 100% effective, then the surviving particles would be only 1S_d_-state atoms that were not removed by laser action.

We collected data for nine different laser frequencies in four sets. Each set involved four distinct frequencies and 21 (or 23, see below) trials at each of these frequencies. In each set, two of the frequencies were always the calculated hydrogen on-resonance frequency at zero laser power (zero detuning) and a far-off-resonance frequency (−200 kHz detuning at 243 nm), as used previously^7^. The other two frequencies in each set were chosen to address various detunings in the neighbourhood of the d–d resonance. The data are summarized in Table 1. The repetition of the points at −200 kHz and zero detuning was intended to address variations in laser power and trapping number between sets. The repetition at + 25 kHz was a check of reproducibility. During the accumulation of data for each set, the four frequencies were interleaved in a varying order and the operators were blinded as to the identity of each frequency setting. The power of the enhancement cavity (about 1 W) was monitored by measuring the transmitted power outside of the vacuum chamber (Fig. 1). Each set was preceded by a thermal cycle of the apparatus to regenerate the cryo-pumping surface.

**Table 1:** Antihydrogen atom counts

	Laser detuning, *D* (kHz)	Number of trials	Atoms lost during laser exposure, *L*	Atoms lost during microwave exposure, *M*	Surviving atoms, *S*	Initially trapped atoms, *N*_i_
Set 1	−200	21	7 ± 7	383 ± 23	504 ± 25	894 ± 35
	−100	21	22 ± 9	415 ± 24	494 ± 24	931 ± 35
	0	21	264 ± 24	423 ± 24	217 ± 16	904 ± 38
	+100	21	75 ± 14	411 ± 23	424 ± 23	910 ± 35
Set 2	−200	21	26 ± 9	394 ± 23	466 ± 24	886 ± 34
	−25	21	113 ± 16	423 ± 24	326 ± 20	862 ± 35
	0	21	219 ± 22	390 ± 23	269 ± 18	878 ± 37
	+25	21	173 ± 20	438 ± 24	296 ± 19	907 ± 37
Set 3	−200	23	8 ± 7	354 ± 22	479 ± 24	841 ± 33
	0	23	303 ± 26	454 ± 25	248 ± 17	1,005 ± 40
	+50	23	176 ± 20	390 ± 23	339 ± 20	905 ± 37
	+200	23	36 ± 11	446 ± 24	459 ± 23	941 ± 35
Set 4	−200	21	7 ± 7	525 ± 26	541 ± 25	1,073 ± 37
	−50	21	86 ± 15	475 ± 25	495 ± 24	1,056 ± 38
	0	21	274 ± 25	480 ± 25	275 ± 18	1,029 ± 40
	+25	21	202 ± 21	516 ± 26	305 ± 19	1,023 ± 38
Total		344	1,991	6,917	6,137	15,045

The integrated number of antihydrogen atoms is listed for each laser detuning (at 243 nm) within each set of trials. The background has been subtracted. Uncertainties quoted are one standard deviation (s.d.) counting errors. We refer to *L* as the ‘appearance signal’; *S* is used to infer the ‘disappearance signal’.

The background-corrected numbers in Table 1 are calculated from raw detector events using the measured, overall efficiencies of the silicon vertex detector. These efficiencies depend on the particular multivariate analysis algorithm that was used to distinguish antiproton annihilations from cosmic rays (Methods) in the relevant time window. The efficiencies and background rates are listed in Table 2.

**Table 2:** Annihilation detector efficiencies and background rates

	Efficiency	Uncertainty	Background rate (10^−3^ s^−1^)	Uncertainty (10^−3^ s^−1^)
Laser exposure (300 s)	0.472	0.001	1.04	0.11
Microwave exposure (32 s)	0.801	0.002	33.0	0.6
Release of surviving atoms (1.6 s)	0.852	0.002	191	1

The detection efficiencies and background rates of the silicon vertex detector, as determined by the multivariate analysis (Methods), are listed for the three observation windows. The 1.6-s window during which the surviving atoms are released extends for 0.1 s after the magnet rampdown is complete.

**Extended Data Fig. 1:**
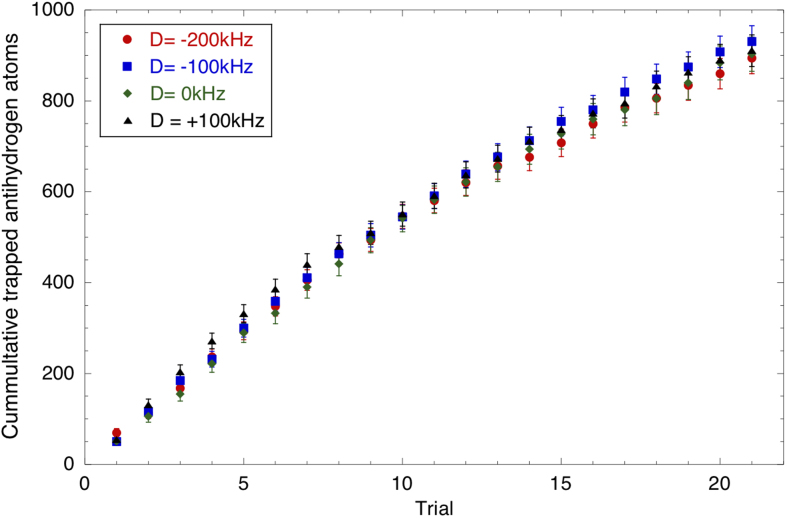
Time evolution of the dataset. The integrated number of atoms is plotted against the trial number for the four detunings *D* (−200 kHz, −100 kHz, 0 kHz and 100 kHz) in set 1. The error bars are 1-s.d. counting uncertainties.

The number of initially trapped atoms *N*_i_ for a trial is unknown a priori, but was typically about 60 at the beginning of a measurement set. In Table 1, the total number of atoms for each group of trials is assumed to be the sum *L* + *M* + *S* of the numbers of atoms lost during laser (*L*) or microwave (*M*) exposure and the number of surviving atoms (*S*) (see Table 1). The trapping rate declined slowly but reproducibly during each set (Extended Data Fig. 1). The third set has 23 trials at each frequency because of a hardware failure in an early block of four trials; extra trials were added to compensate for the excluded data.

**Fig. 3:**
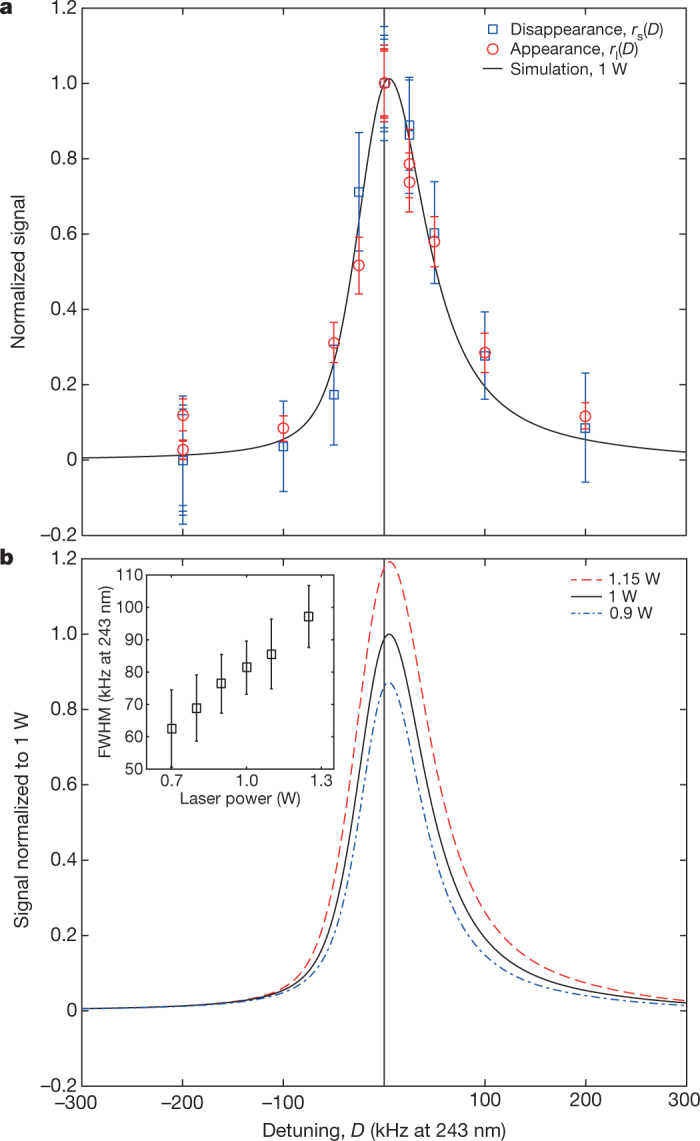
Spectral line of antihydrogen. **a**, The complete dataset, scaled as described in the text. The simulated curve (not a fit, drawn for qualitative comparison only) is for a stored cavity power of 1 W and is scaled to the data at zero detuning. ‘Appearance’ refers to annihilations that are detected during laser irradiation; ‘disappearance’ refers to atoms that are apparently missing from the surviving sample. The error bars are 1-s.d. counting uncertainties. **b**, Three simulated line shapes (for hydrogen) are depicted for different cavity powers to illustrate the effect of power on the size and the frequency at the peak. The width of the simulated line (FWHM) as a function of laser power is plotted in the inset.

To examine the general features of the measurement results, we plot (Fig. 3a) the four datasets on one graph by using a simple scaling. The points at zero (on-resonance) and −200-kHz detuning (at which no signal is expected^7^), repeated for each set, are used for the scaling. For the laser exposure (‘appearance’) data, we define a scaled response at detuning *D* within each set: *r*_l_(*D*) = *L*(*D*)/*L*(0). Similarly, for the surviving population (‘disappearance’ data), we use *r*_s_(*D*) = [*S*(−200 kHz) − *S*(*D*)]/[*S*(−200 kHz) − *S*(0)]. The uncertainties shown are due to Poissonian counting errors only. For comparison, we also plot the results of a simulation^19^ based on the expected behaviour of hydrogen in our trap for a cavity power of 1 W, scaled to the zero-detuning data point. We see that the peak position and the width of the scaled spectral line are consistent with the calculation for hydrogen and that the experiment generally reproduces the predicted asymmetric line shape. There is also good agreement between the appearance and disappearance data (Fig. 3a).

The simulation involves propagating the trapped atoms in an accurate model of the magnetic trap. When an atom crosses the laser beam, which has a waist of 200 μm at the cavity centre, we calculate the two-photon excitation probability, taking into account transit-time broadening, the a.c. Stark shift and the residual Zeeman effect. The simulation determines whether excited atoms are lost owing to ionization or to a spin-flip event. The variable input parameters for the simulation are the cavity power and the laser frequency. The modelled response is asymmetric in frequency owing to the residual Zeeman effect^19^. The width of the line, for our experimental parameters, is dominated by transit-time broadening, which contributes about 50 kHz full-width at half-maximum (FWHM) at 243 nm. For 1 W of cavity power, the a.c. Stark shift is about 2.5 kHz to higher frequency and the ionization contributes about 2 kHz to the natural line width.

To make a more quantitative comparison of the experimental results with the expectations for hydrogen, it is necessary to scrutinize differences between the four datasets. The overall response should be linear in the number of atoms addressed, so it is possible to normalize for this. However, the line width depends on the stored power in the cavity, as does the frequency of the peak (Fig. 3b). The cavity power is difficult to measure in our geometry because the amount of transmitted light depends sensitively on the small transmission from the output coupler (about 0.05%) and on absorption in the optical elements through which the transmitted light exits (Fig. 1). We observe that the transmitted power can degrade, owing to accumulated ultraviolet damage to the window and mirror substrate, whereas the finesse of the cavity does not change.

A modelling approach that self-consistently accounts for fluctuations in experimental parameters is a simultaneous fit in which we allow the four sets to have distinct powers (*P*_1–4_), but the same frequency shift with respect to the hydrogen calculation (Methods). We require that the average powers for the appearance and disappearance data within a set are the same. We find the parameters that best reproduce the data to be: *P*_1_ = 1135(50) mW, *P*_2_ = 904(30) mW, P_3_ = 1123(43) mW, *P*_4_ = 957(31) mW and δ*f* = −0.44 ± 1.9 kHz, where δ*f* is the difference (at 243 nm) between the resonant frequency inferred from the fit and the resonant frequency of hydrogen expected for our system, both at zero power. The uncertainties represent the 68% confidence interval of a least-squares fit and do not take into account systematic uncertainties. The fit uses the five variables identified above, and the individual data points at each frequency are weighted by their Poissonian counting errors. We include an uncertainty of 3.8 kHz (Table 3) in the final resonance frequency to represent statistical and curve-fitting uncertainties.

**Table 3:** Summary of uncertainties

Type of uncertainty	Estimated size (kHz)	Comment
Statistical uncertainties	3.8	Poisson errors and curve fitting to measured data
Modelling uncertainties	3	Fitting of simulated data to piecewise-analytic function
Modelling uncertainties	1	Waist size of the laser, antihydrogen dynamics
Magnetic-field stability	0.03	From microwave removal of 1S_c_-state atoms (see text)
Absolute magnetic-field measurement	0.6	From electron cyclotron resonance
Laser-frequency stability	2	Limited by GPS clock
d.c. Stark shift	0.15	Not included in simulation
Second-order Doppler shift	0.08	Not included in simulation
Discrete frequency choice of measured points	0.36	Determined from fitting sets of pseudo-data
Total	5.4	

The estimated statistical and systematic errors (at 121 nm) are tabulated.

Considering systematic effects, the microwave removal procedure for the 1S_c_-state atoms provides a reproducibility check on the strength of the magnetic field at the centre of the trap. At the beginning of each data-taking shift, the magnetic field of the external solenoid magnet was reset to a standard value using an electron cyclotron resonance technique^16^. For the complete dataset, we find that the variations in the magnetic field at the minimum field of about 1 T are about 3.2 × 10^−5^ T (1 s.d.). This corresponds to a resonance frequency shift^19^ of only about 15 Hz at 243 nm for the d–d transition. (At 1 T, the c–c transition is about 20 times more sensitive to magnetic field shifts, which is why the d–d transition is more attractive here.) The laser frequency was tuned with respect to the minimum of the magnetic well, such that the resonance condition should be met in the centre of the trap for zero detuning in the limit of zero laser power. The accuracy of the magnetic-field determination corresponds to an uncertainty of 300 Hz in the 243-nm laser frequency.

Including all of the statistical and systematic uncertainties that we have identified (Table 3, for 121 nm), our fit of the experimental data to the hydrogen model yields

$${f}_{{\rm{d}}-{\rm{d}}}=\mathrm{2,466,061,103,079.4}(5.4)\,{\rm{k}}{\rm{H}}{\rm{z}}$$

The value (Methods) for hydrogen calculated at the minimum field in our system (1.03285(63) T) is

$${f}_{{\rm{d}}-{\rm{d}}}=\mathrm{2,466,061,103,080.3}(0.6)\,{\rm{k}}{\rm{H}}{\rm{z}}$$

where the uncertainty is determined by the experimental error in measuring the field.

Owing to the motion of the antihydrogen atoms in the inhomogeneous trapping field, this comparison is necessarily model-dependent. We therefore conclude that the measured resonance frequency for this transition in antihydrogen is consistent with the expected hydrogen frequency to a precision of about 2 × 10^−12^. Although the precision of our measurement is still a few orders of magnitude short of the state of the art with a cold hydrogen beam^8^, the modern frequency reference permits the accuracy of our experiment to exceed that achieved with trapped hydrogen^20^ as recently as the mid-1990s. We used a total of about 15,000 antihydrogen atoms to obtain this result, compared to 10^12^ trapped atoms in the analogous matter experiment. Our dataset was accumulated over a period of ten weeks, illustrating that the antihydrogen trapping procedure is robust and that systematic effects are manageable. ALPHA’s emergent antihydrogen production, storage and detection techniques, together with advances in ultraviolet laser technology and frequency metrology, pioneered by Hänsch and colleagues, enable precision anti-atom spectroscopy.

Precision experiments at the antiproton decelerator have recently constrained the properties of the antiproton through studies in Penning traps^21, 22^ or with antiprotonic helium^23^. For example, the antiproton charge-to-mass ratio is known to agree with that of the proton to 69 parts per trillion^21^, equivalent to an energy sensitivity of 9 × 10^−27^ GeV. The ratio of the antiproton mass to the electron mass has been shown to agree with its proton counterpart^23^ to 8 × 10^−10^, and antihydrogen has been shown to be neutral^24^ to 0.7 parts per billion. Our measurement of antihydrogen probes different and complementary physics at a precision of a few parts per trillion, or an energy level of 2 × 10^−20^ GeV. This already exceeds the precision (4 × 10^−19^ GeV) in the mass difference of neutral kaons and antikaons^25^, which has long been the standard for particle-physics tests of charge–parity–time invariance.

Near-term improvements in the ALPHA-2 apparatus will include a larger waist size for the radiation in the optical cavity to reduce transit-time broadening, operation at lower magnetic fields and operational improvements to accelerate data acquisition and to reduce statistical uncertainties. Future measurements will require an upgrade to our frequency reference to exceed a fractional precision of 8 × 10^−13^ (Methods). The rapid progress detailed here confirms that, in principle, there is nothing to prevent the achievement of hydrogen-like precision in antihydrogen and the associated very sensitive test of charge–parity–time symmetry in this system.

## Methods

### Time evolution of the dataset

The time evolution of the atoms detected in one of the datasets is depicted in Extended Data Fig. 1.

### Laser system for 243-nm light

A Toptica TA-FHG pro laser system uses a pair of frequency-doubling cavities to generate 150 mW of 243-nm light from a 972-nm extended cavity diode laser (ECDL). The 243-nm beam is mode-matched to the 1S–2S enhancement cavity and sent along a 7-m-long path with active beam-pointing stabilization between the laser laboratory and the ALPHA-2 apparatus. The enhancement cavity is locked to the laser frequency using a single piezoelectric actuator located behind the output coupler mirror^26^ to feedback on an error signal generated via the Pound–Drever–Hall technique^27^. The light transmitted through the cavity is monitored using a photodiode that is located outside the vacuum system. The cavity has a measured finesse of 250 and achieves a circulating power of approximately 1 W.

The 972-nm ECDL is frequency-stabilized (also using the Pound–Drever–Hall technique) to a Menlo Systems ultralow-expansion cavity via an acousto-optic modulator, which shifts the light from the 1S–2S transition frequency of the laser to the closest resonance of the ultralow-expansion cavity. The resonance frequency of the cavity is monitored continuously using a Menlo Systems femtosecond frequency comb, which is referenced to atomic time using a K + K Messtechnik GPS-disciplined quartz oscillator.

The measured difference between the ultralow-expansion resonance frequency and a comb line with a known frequency is fed forward to the control of the acousto-optic modulator with an averaging time of 20 s to remove long-term drifts. The uncertainty of the frequency difference over the 20-s averaging period corresponds to an Allan deviation^28^ of 75 Hz at 972 nm (300 Hz at 243 nm). One of the frequency-comb counters is used to measure the signal from a Symmetricom CS4000 caesium clock to confirm correct operation of the quartz oscillator and the radio-frequency chain of the frequency comb. The count reaches a fractional Allan deviation of 8 × 10^−13^ after 1,000 s of averaging, which corresponds to fluctuations of 250 Hz at 972 nm (1 kHz at 243 nm).

An independent, identical, 972-nm ECDL frequency stabilized to an independent, identical, ultralow-expansion cavity is used to evaluate the short-term line width of the spectroscopy laser. The beat note generated between the two 972-nm lasers has a spectrum composed of individual lines, each with a line width of less than 1 Hz, within a 300-Hz (1.2 kHz at 243 nm) FWHM Gaussian envelope. The source of the broadening is thought to be acoustic noise within the laser laboratory; work is ongoing to reduce the broadening effect.

### Suppression of cosmic-ray background

To determine the signal events in the (a) 1.6-s, (b) 32-s and (c) 300-s observation windows, we require three different suppression techniques. (The 1.6-s window extends to 0.1 s after the magnet rampdown is complete.) We tune the multivariate analysis (MVA) for each of the three windows to optimize the statistical significance of the estimated signal. Annihilation events are distinguished from background events (primarily cosmic rays) by their distinctive topologies. Fourteen selection variables that are sensitive to the difference between annihilation and background events were used as inputs to an MVA package^18^. The variables included are: (i) the total number of channels registering ‘hits’ by charged particles; (ii) the radial coordinates of the reconstructed annihilation vertex; (iii) the sum of the squared residual distances of hits from a fitted straight line; six topological variables (iv–ix); and five additional variables (x–xiv). The topological variables are: (iv) a sphericity variable; (v) the cosine of the angle between the event axis and the detector axis; (vi) the angle between the event axis and the vertical direction in the *x*–*y* plane; (vii) the number of reconstructed tracks; (viii) the number of three-hit combinations used as track candidates; (ix) the distance of closest approach of the tracks. The additional variables are: (x) the minimum and (xi) mean values of the track radius in canonical form; (xii) the minimum and (xiii) mean values of the pitch of the helical track in canonical form; and (xiv) an integer sum of the sense of curvature (left = −1 or right =  + 1) for all of the tracks in the event.

The signal data and background data used for MVA training and testing comprise a set of 580,846 annihilation events and 3,740,613 background events. The signal events were produced during antiproton and positron mixing in the apparatus and contain less than 1% background. Background events were collected during times when there were no antiprotons in the apparatus.

*The 1.6-s observation window*. A classifier cut was chosen to optimize the significance for an expected 200 counts of signal and 350 counts of background. The analysis gives a background rate of 0.191 ± 0.001 s^−1^ and an efficiency of 0.852 ± 0.002 (statistical error only) annihilations per detector trigger.

*The 32-s observation window*. The analysis was chosen to optimize the significance for an expected 400 counts of signal and 3,500 counts of background. The analysis gives a background rate of 0.033 ± 0.0006 s^−1^ and an efficiency of 0.801 ± 0.002 (statistical error only) annihilations per detector trigger.

*The 300-s observation window*. A classifier cut was chosen to optimize the significance for an expected 250 counts of signal and 330,000 counts of background. The analysis gives a background rate of 0.0010 ± 0.0001 s^−1^ and an efficiency of 0.472 ± 0.001 (statistical error only) annihilations per detector trigger.

### Fitting the data using the hydrogen simulation

The build-up of laser power in the enhancement cavity is one of the primary experimental parameters that influence the data in Table 1. The main effect of a change in laser power is on the amplitude of the measured line, but there is also an effect on the peak position through the a.c. Stark shift and on the line width owing to depletion effects. In our set-up, there is considerable uncertainty in measuring the absolute intra-cavity laser power; relative measurements show that although the constancy of laser power within any single measurement set is good, there are variations between the sets.

To reflect this experimental reality in our analysis of the data, the *χ*^2^ statistic for the full dataset is minimized with respect to a function that, aside from an overall frequency shift, allows a unique laser power in each set and incorporates the effects of those laser powers on the amplitude, line width and line centre based on the simulation of hydrogen in our experiment.

**Extended Data Fig. 2:**
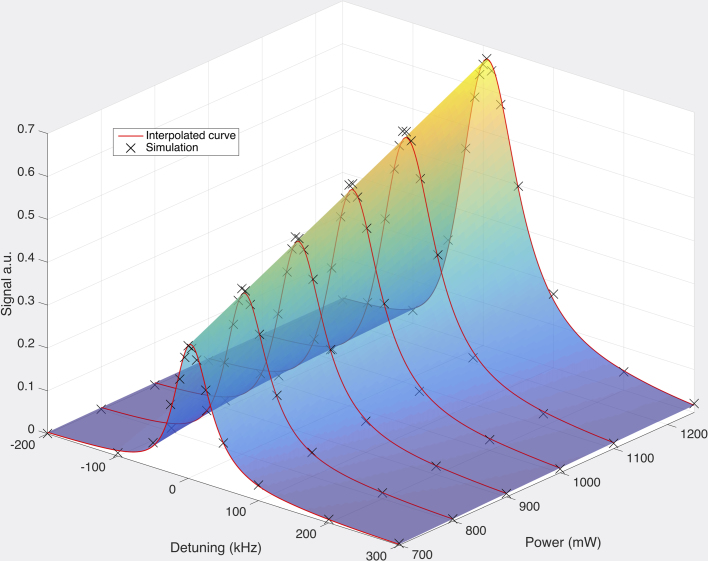
Simulation fitting functions. The points (crosses) from the numerical simulation are plotted for various cavity powers. The solid lines represent fits to the simulation by a piecewise-analytic function. The coloured surface represents the interpolation used to fit the experimental data.

The construction of the fit function therefore starts by running a detailed simulation of hydrogen in the ALPHA-2 magnetic trap for an array of input laser powers and frequencies that spans the experimentally relevant values, in this case from −200 kHz to + 300 kHz in laser detuning and from 0.7 W to 1.25 W in laser power. We simulate a total of 365,000 atoms in this array, after which we interpolate to obtain continuous values in both laser detuning and power. The interpolation in power is a linear regression at each detuning in the array, based on the observed linear behaviour. For interpolation in detuning, a fit to a piecewise-analytic function that provides a good approximation to the simulation data is used. An error associated with this fit is included in Table 3. The discrete simulated points and the smooth interpolation are plotted in Extended Data Fig. 2.

### Calculation of the resonant frequency for hydrogen

The frequency *f*_d–d_ is calculated from corrections to the centroid-to-centroid frequency *f*_1S2S_:

$$\begin{array}{c}{f}_{{\rm{d}}-{\rm{d}}}(B)={f}_{1{\rm{S}}2{\rm{S}}}-\frac{1}{4}[{f}_{{\rm{H}}{\rm{F}}}(1)-{f}_{{\rm{H}}{\rm{F}}}(2)]+[{\mu }_{{\rm{e}}}(2)-{\mu }_{{\rm{e}}}(1)]\frac{B}{h}\\ \phantom{\rule{27pt}{0ex}}-[{\mu }_{{\rm{p}}}(2)-{\mu }_{{\rm{p}}}(1)]\frac{B}{h}+{(\frac{m}{\mu })}^{3}\frac{13{e}^{2}{a}_{0}^{2}}{4mh}{B}^{2}\end{array}$$

where *h* is Planck’s constant, *f*_HF_(*n*) is the hyperfine splitting of the state with principle quantum number *n*, *μ*_e_ and *μ*_p_ are the magnitudes of the magnetic moments of the electron and proton, respectively, *μ* is the reduced mass of the electron, *m* is the electron mass, *e* is the fundamental charge, *a*_0_ is the Bohr radius for an infinite-mass nucleus and *B* is the magnetic field.

The first correction describes the difference in the hyperfine splittings of the 1S and 2S states. The second (third) correction describes the difference in the magnetic moment of the electron (proton) in these states. The fourth correction describes the difference in the diamagnetic shift.

The magnetic moment of the bound electron is (equation (84))^29^

$${\mu }_{{\rm{e}}}(n)={\mu }_{{\rm{e}}}^{{\rm{free}}}\left[1-\frac{{\alpha }^{2}}{3{n}^{2}}+\frac{{\alpha }^{4}}{2{n}^{3}}\left(\frac{1}{2n}-\frac{2}{3}\right)+\frac{{\alpha }^{3}}{4{\rm{\pi }}{n}^{2}}+\frac{{\alpha }^{2}}{2{n}^{2}}\frac{m}{M}\right]$$

where *α* is the fine-structure constant, ${\mu }_{{\rm{e}}}^{{\rm{free}}}$ is the free-electron dipole moment and *M* is the proton mass; the dependence on *n* is described elsewhere^30, 31^. The magnetic moment of the bound proton is (equation (87))^29^

$${\mu }_{{\rm{p}}}(n)={\mu }_{{\rm{p}}}^{{\rm{free}}}\left[1-\frac{{\alpha }^{2}}{3{n}^{2}}+\frac{{\alpha }^{2}}{6{n}^{2}}\frac{m}{M}\left(\frac{3+4\times 1.793}{1+1.793}\right)\right]$$

where ${\mu }_{{\rm{p}}}^{{\rm{free}}}$ is the free-proton dipole moment.

Using current CODATA values^32^ for the fundamental constants, the frequency is

$$\begin{array}{c}{f}_{{\rm{d}}-{\rm{d}}}={f}_{1{\rm{S}}2{\rm{S}}}-\mathrm{310,712.229}\,{\rm{k}}{\rm{H}}{\rm{z}}+186.071B\,{\rm{k}}{\rm{H}}{\rm{z}}{{\rm{T}}}^{-1}\\ \,-0.283B\,{\rm{k}}{\rm{H}}{\rm{z}}{{\rm{T}}}^{-1}+387.678{B}^{2}\,{\rm{k}}{\rm{H}}{\rm{z}}{{\rm{T}}}^{-2}\end{array}$$

### Sample size

No statistical methods were used to predetermine sample size.

### Data availability

The datasets generated and analysed during this study are available from the corresponding author on reasonable request.

## Online content

Any Methods, including any statements of data availability and Nature Research reporting summaries, along with any additional references and Source Data files, are available in the online version of the paper at 10.1038/s41586-018-0017-2.

## Extended data

are available for this paper at 10.1038/s41586-018-0017-2.
